# The role of birth companionship in women’s experiences of mistreatment during childbirth and postpartum anxiety and depression: An analysis of a cross-sectional survey

**DOI:** 10.1371/journal.pgph.0004030

**Published:** 2025-07-30

**Authors:** Waqas Hameed, Bushra Khan, Bilal Iqbal Avan

**Affiliations:** 1 Department of Community Health Sciences, Aga Khan University, Karachi, Pakistan; 2 Department of Psychology, University of Karachi, Karachi, Pakistan; 3 Department of Population Health, London School of Hygiene and Tropical Medicine, London, United Kingdom; Curtin University, AUSTRALIA

## Abstract

There is limited evidence on how companionship affects women’s experiences of mistreatment during childbirth and their mental health. We assessed the characteristics of birth companionship during intrapartum care and examine its associations with women’s experiences of mistreatment and symptoms of postpartum anxiety and depression. We analysed cross-sectional data from 314 women who gave birth at six public health facilities in rural Sindh, Pakistan. These women were interviewed at 42 days postpartum about their childbirth experiences and symptoms of anxiety and depression. Multivariable regression models and Path analysis were used for data analysis. Most women (83.1%) had a full-time companion in the labor room, with in-laws (44.6%) being the most common. Higher levels of companion support were associated with lower levels of mistreatment, particularly regarding non-confidential care, lack of supportive care, and ineffective communication. Women receiving low companion support were 2.86 (95% CI 1.52 – 5.39) times more likely to experience postpartum anxiety and depression compared to those receiving high support. Informational support emerged as the strongest protective factor against both mistreatment and symptoms of postpartum anxiety and depression. Path analysis revealed that both the overall measure of companion support and informational support had direct and indirect effects on postpartum anxiety and depression, mediated by experiences of mistreatment during childbirth. In contrast, emotional support demonstrated only an indirect effect through mistreatment, while instrumental support showed only a direct effect on postpartum anxiety and depression symptoms. Birth companionship, especially informational support, plays a crucial role in reducing mistreatment during childbirth and improving maternal mental health outcomes. These findings underscore the need for supportive policies and health system interventions that actively encourage the engagement of companions from a woman’s personal network during labour and childbirth. Future research should explore strategies to optimise the role of birth companions in promoting respectful care and maternal well-being.

## Authors’ summary

We assessed whether having a companion during childbirth affects women’s experiences of mistreatment and their mental health after giving birth. Data on 314 women were analysed who gave birth in six public hospitals in rural Sindh, Pakistan. Women were interviewed 42 days after childbirth about their experiences and mental health. The findings show that 83.1% of women had a companion during labour, most often an in-law, family member, or friend. Women with lesser companion support were more likely to experience mistreatment, especially lack of privacy, poor communication, or unsupportive care. Moreover, women with low companion support were nearly three times more likely to experience postpartum anxiety and depressive symptoms. Informational support was particularly showed stronger relationship with reduction in mistreatment and improving mental health. We also found that overall support from birth companions, particularly informational support, directly reduced anxiety and depression symptoms and indirectly did so by reducing mistreatment during childbirth. These results highlight the importance of birth companionship, especially in providing information and emotional support, to reduce mistreatment and promote better mental health for mothers. Policies and healthcare practices should encourage women to have trusted companions during childbirth to ensure respectful care and improve well-being.

## Introduction

One-third of women perceive their childbirth experience as traumatic [1], a significant event that can profoundly change a woman’s life and leave enduring memories. In low- and middle-income countries (LMICs), about 1 in 5 women suffer from common perinatal mental disorders [2]. These psychological distresses during labour make women uniquely vulnerable to environmental influences such as unfamiliar personnel, medicalised procedures, and other conditions, leading them to prefer the presence of someone familiar and comfortable during labour and childbirth [3]. Yet, health systems in LMICs primarily focus on physical health, often deprioritising or neglecting social and emotional needs [4]. Unfortunately, women are often faced with disrespectful and abusive behaviours during childbirth in facility-based settings [5]. These woeful experiences are likely to have both short- and long-term adverse effects, such as dissatisfaction with maternity care, feelings of re-traumatisation, postpartum depression (PPD), and challenges in maternal-infant bonding [3,4].

The World Health Organization (WHO) has strongly recommended the presence of companions of choice during labour and childbirth in their recent guidelines on maternal and newborn care [6–8]; birth companions are also considered as an integral component of respectful maternity care [9]. A review of qualitative studies highlighted birth companions could play a diverse tangible supportive roles, that include emotional support (e.g., reassurance and praise), informational support (e.g., updates on labour progress and coping techniques), instrumental support (e.g., massage, encouraging mobility), and advocacy (e.g., helping the woman articulate her wishes to others) [10]. A 2017 Cochrane systematic review showed that supportive care during childbirth either from personal companion or professional team improves a range of maternal and infant outcomes. These include a shorter duration of labour, higher perceived control over birth, lower perceived labour pain, a greater likelihood of spontaneous vaginal birth, and increased maternal satisfaction with childbirth services [3]. Despite promising evidence and recommendations from the WHO, the coverage of companionship during labour and childbirth varies considerably across countries, ranging from 4% to 94% [11].

While several studies have sought to enhance understanding of birth companionship, including general perceptions, associated factors, and its impact on maternal outcomes, limited evidence is available on how companionship affects women’s experiences of mistreatment during childbirth and their mental health. Few studies [12–14] came out recently and all of them consistently showed that presence of birth companion reduces the likely of mistreatment. For instance, in Guinea, the absence of labour companionship increased the odds (adj. OR=3.6) of any physical abuse, verbal abuse, or stigma or discrimination and non-consented vaginal examinations (adj. OR = 3.2). Similarly, in Ghana, it is associated with non-consented vaginal examinations (adj. OR = 2.3) and poor communication (adj. OR=2.0) [15]. However, none of them examined the characteristics of birth companionship and its relationship with mistreatment [12–14]. Furthermore, there is research examining the relationship between birth companions and maternal mental health. Most of the existing research has focused on the presence of a personal birth companion rather than the impact of the characteristics of that companionship on mental health [16–19]. For example, Iranian primipara women without a birth companion are 3.3 times more likely to experience traumatic childbirth compared to those with a birth companion [16]. Similarly, few experimental studies also demonstrated that presence of birth companion significantly decrease maternal anxiety [20,21]. This study seeks to address existing gaps by: (a) assessing the characteristics of birth companionship during intrapartum care; (b) examining the associations between birth companionship, women’s experiences of mistreatment, and postpartum anxiety and depression symptoms; and (c) exploring whether the relationship between birth companionship and symptoms of postpartum anxiety and depression is mediated by women’s experiences of mistreatment.

### Research question

What are the characteristics of birth companionship during intrapartum care, and how they are associated with women’s experiences of mistreatment and postpartum anxiety and depression symptoms?

### Country context

Pakistan has a population of 245 million [22], with two-thirds residing in rural areas [23]. Healthcare in Pakistan is delivered through a three-tiered health system: primary, secondary and tertiary. The current research focused on secondary-level healthcare where inpatient childbirth facilities are formally introduced through maternity ward and labour room services. Specialist consultation and hospital admissions fall into this category. It includes two types of health facilities: district (District health quarter Hospitals–DHQ) and sub-district level (Tehsil Head Quarter—THQs). Pakistan has one of the highest levels of maternal [24] and neonatal mortality in the region. While 7 in 10 women give birth in facility-based settings [23], more than 95% report experiencing at least one instance of disrespect and abuse (D&A) during labour and delivery [25,26]. Estimates suggest that 1 in 4 birthing women suffer from perinatal depression—a rate higher than the pooled estimate for low- and middle-income countries (LMICs) [27]. The choice of personal birth companions, particularly husbands, is often restricted in hospitals due to barriers such as the use of traditional methods by companions, social norms, inadequate facility layouts, cultural diversity, and hospital policies [28–30]. To our knowledge, no study in Pakistan has assessed the relationship between birth companionship, mistreatment, and maternal mental health.

## Methods

### Ethics statement

The study protocol, the informed consent forms, and other appropriate documents were approved by the Ethics Review Committee of the Aga Khan University (Ref. ID: 2019-1683-5607) and Research Ethics Committee of the London School of Hygiene & Tropical Medicine (Ref. ID: 17886). The study has been registered with clinicaltrials.gov (registration number: NCT05146518). Please see S1 Checklist Inclusivity Checklist.

### Design and settings

This study analyses cross-sectional endline survey data collected between 13th September and 11th December 2021, as part of a larger experimental study conducted in two rural districts of southern Sindh, Pakistan [31]. The broader study aimed to promote supportive and respectful maternity care (S-RMC) in six secondary-level public healthcare facilities. However, the analysis presented here does not assess the effects of the intervention. Instead, it only utilises the endline data to explore other specific research questions within the broader context of the study.

The health facilities provided at least standard basic emergency obstetric and newborn care services. Deliveries ranged from 40 to 300 per month at each facility. The study protocol and the results of primary analysis has been published [31,32]. Briefly, S-RMC intervention targeted skill enhancement of maternity teams and systemic improvements for better governance and accountability in health facilities. It involved comprehensive team training in respectful and psychosocial care, followed by the implementation of practices like vulnerability screening and provision of respectful care and psychosocial support during maternity care. Engagement of birth companion from women’s personal network like mother, sister etc. was an integral component of psychosocial support [33]. The intervention also introduced a feedback system through complaints and exit interviews, and administrative measures like S-RMC data review for performance improvement, staff recognition, and professional development. A mental health first-aider supervised these activities, supported by the research team to ensure effective integration of S-RMC practices [31].

### Data collection and management

A total of 314 women were interviewed. Every woman who had given birth at the health facility (study site) during the period of data collection, regardless of maternal characteristics or birth outcome. Women who were admitted but later referred to other hospitals due to pregnancy complication were not included. These women were initially contacted at health facilities and subsequently interviewed in their homes regarding their childbirth experiences, 42 days after delivery to minimise courtesy bias due to the proximity of maternity service providers. A consecutive sampling technique was used to recruit participants for the study. All women who delivered at the selected facilities during the data collection period were asked to participate in the survey until the desired sample size was achieved. The distribution of the sample across the health facilities was proportional to their childbirth caseloads. Details of sample size calculations are published elsewhere [31]. Data collection was carried out simultaneously at all six facilities. Only those women who consented to interviews at both the health facility and their homes six weeks postpartum were included in the survey. Women living outside the study district or in remote rural areas were excluded due to logistical difficulties and potential security concerns for the data collectors (Table 1). The lost-to-follow-up was less than 5%, with primary reasons being migrated/shifted and or the data collection team was unable to locate the household. Data collection was conducted electronically using tablets, utilising Epicollect5 software which included built-in validation checks to maintain data quality. Data collectors were trained on the survey questionnaire to minimise interview bias. Routine assessments of data quality were performed through simple frequency counts and cross-classification analysis. All participating women provided written informed consent.

**Table 1:** Eligibility criteria for study participants.

Inclusion	Exclusion
All women who give birth at the study sites during the data collection period, regardless of maternal demographic or clinical characteristics, or birth outcomes	Women residing outside the study district or in remote rural areas that are inaccessible due to logistical difficulties and potential security risks for data collectors
Women who provide written informed consent for both facility-based and follow-up (42-day home-based) interviews.	Women who were admitted to the study site but subsequently referred to another non-study facility for childbirth.
	Women who died during labour, childbirth, or within the immediate postpartum period (before being eligible for the 42-day follow-up).
	Women unable to communicate in the language used for data collection

### Measures

#### Dependent variables.

We used two outcome variables: women’s experiences of mistreatment (continuous scale, 0–100) and postpartum anxiety and depression symptoms (binary: no/yes). An open-access, community-based structured instrument, developed by an international group, was adapted to document experiences of mistreatment [34]. We utilised the WHO’s mistreatment framework to develop a composite measure for overall mistreatment and specific types. The overall mistreatment score was derived from 53 binary items, scoring “1” for experienced mistreatment and “0” for none, with total scores ranging from 0 to 53 and linearly transformed to a 0–100 scale. This score reflects the cumulative level of mistreatment during maternity care, where higher scores indicate women experienced greater number of mistreatment manifestations. We applied the same scoring method for each mistreatment type, also scaled from 0 to 100. The Kuder Richardon’s value of internal consistency [35] for mistreatment measures were: overall mistreatment (0.77), physical abuse (0.51), verbal abuse (0.64), non-confidential care (0.32), ineffective communication (0.67), lack of supportive care (0.69), lack of professional standards (0.32), and health system constraints and conditions (0.52). The symptoms of anxiety and depression was screened using Patient Health Questionnaire – 4 at 42 days postpartum [36]. Standard cut-off was used to create a binary measure.

#### Independent variable.

Birth companions referred to individuals chosen by a woman from her personal network—such as her husband, mother, or a friend—to provide support during labour and childbirth.

We used two main independent variables: quality of support and type of supportive provided by the birth companion during the stay in the health facility for childbirth. We created a composite score to reflect quality of companion support based on nine binary items that were classified into three categories: A) Emotional support: (i) console through touch and reassuring words, (ii) distract by talking about any subject to ease anxiety or pain, (iii) avoid doing or saying anything that may hurt or upset; B) Instrumental support: (i) Help her adopt an alternative position to ease pain, (ii) Maintain privacy, (iii) Assist to ambulate during labour; C) Informational support: (i) encourage and/or remind her of the breathing exercise, (ii) update women about current condition, (iii) support regarding nutritional and medicinal intake.. Items for companion support were based on WHO recommendations [37], cultural practices and suggestions from maternity staff working in labour room settings. These items or indicators were identified via systematic and iterative process and their content and cultural relevance were ensured [33].

The overall composite score of quality of companion support was constructed by summing the scores of 9 binary items, where “1” indicates “yes,” and “0” indicates “no”. The total raw score ranged from 0 to 9, with a higher score indicating a higher level of support provided to the women during maternity care. The Kuder Richardon’s value of internal consistency for the overall measure was 0.75. The overall measure of companionship was categorised into: low (0–5), moderate (6–7), and high (8–9) level of support. For the measure of support type, women who reported receiving all three forms of support within a given dimension (e.g., emotional, instrumental, or informational) were coded as “1,” while those who received less were coded as “0.”

### Statistical analysis

The statistical analyses were performed in multiple stages in accordance with the study objectives. First, we used descriptive analysis to describe the characteristics of study population, and level and kind of support provided by the birth companion to women. Second, we used simple and multivariable regression models to determine the relationship between measures of birth companion and mistreatment and symptoms of postpartum anxiety and depression. Keeping in view of the type of outcomes variable (continuous or binary), linear and logistic regression techniques were applied, as appropriate. To assess the relationship between primary exposure and outcome, we fitted both unadjusted and adjusted models. First, we ran unadjusted models to examine crude associations. Subsequently, we ran multivariable models that adjusted for covariates that were either statistically or biologically plausible [38–40] which included: age, order of pregnancy, ethnicity, education, household poverty, level of women’s involvement in household decision-making, mode of birth, and sex of the index baby. Finally, we employed path analysis to examine whether or not the relationship between birth companionship and symptoms of postpartum anxiety and depression is mediated by experiences of mistreatment during childbirth. We ran separate multivariable regression models to examine the relationship on each path [41]: linear regression (effect of companion support and mistreatment) and logistic regression (effect of companion support and mistreatment on symptoms of postpartum anxiety and depression). All regression models accounted for potential covariates/confounders that were either statistically or biologically plausible. The identification of potential covariates/confounders for each path was guided by the published systematic reviews [42–45] and these are mentioned in the footnotes of Fig 2A-D. The estimated coefficients from separate models were then put together in a path diagram (see Fig 2A-D). The same procedure was applied for all the overall measure of birth companionship and each of the three types of companion support. It is pertinent to note that we used discrete companionship variables in these models, with reverse coding to indicate the lack of companion support. In accordance with the objective, the findings of only three primary variables (companionship, mistreatment, and anxiety and depression symptoms) were presented in the path diagram. The effect of possible clustering due to the correlation within health facilities was taken into account by estimating cluster-adjusted standard errors using *svy* command in Stata. Stata version 18.0 (StataCorp, College Station, TX) was used for all analyses. P-value of <0.05 were considered significant.

**Inclusivity in global research:** Additional information regarding the ethical, cultural, and scientific considerations specific to inclusivity in global research is included in the Supporting Information (S1 Checklist).

## Results

Table 2 describes the socio-demographic and reproductive characteristics of the study participants. The mean age of the women was 29.7 (±5.2) and about one-fourth had at least one functional disability. The majority were Sindhi (93.0%) spoken, had no formal education (81.2%), and about half of them were living on less than $1.25 a day. Regarding reproductive characteristics, 84.4% were primigravida, 93.0% received antenatal care, and 86.0% had vaginal births.

**Table 2:** Socio-demographic and reproductive characteristics of study participants.

Characteristics	n	%
**Socio-demographics**		
Women’s age		
Mean (± SD)	29.7	5.2
Mother tongue		
Sindhi	292	93.0
Urdu, Punjabi, Balochi, Pushto, Saraiki, Brohi, Gujrati	22	7.0
Women education		
None/illiterate	255	81.2
Attended any formal education	59	18.8
Poverty		
Women that live on less than $1.25 a day	50.9	15.2
Functional disability		
None	246	78.3
Any one or more disability or at risk of disability	68	21.7
Women’s involvement in household decision making (Score range: 0–14)		
Mean (± SD)	6.0	5.1
**Reproductive characteristics**		
Primigravida		
Yes	265	84.4
No	49	15.6
Received antenatal care for index childbirth		
Yes	292	93.0
No	22	7.0
Mode of birth for current pregnancy		
Caesarean section	44	14.0
Vaginal birth	270	86.0
Sex of baby at most recent birth		
Boy	162	51.6
Girl	152	48.4

Table 3 details the presence and role of personal companions during labour and childbirth. An overwhelming majority (98.7%) had a companion at some point during their hospital stay. Companions were most commonly present after childbirth (98.4%), during labour (93.6%), and during childbirth itself (85.7%). Most women (83.1%) were allowed a companion full-time in the labour room. In the labour room, the majority of women were accompanied by their in-laws (44.6%), followed by family members (26.1%), and friends (15.0%).

**Table 3:** Companion support during labour and childbirth (n = 314).

Characteristics	n	%
**Women had birth companion at any point during her stay in the hospital**		
Yes	310	98.7
No one accompanied the woman	4	1.3
**When was the companion present with you?**		
During labour before childbirth	294	93.6
During childbirth	269	85.7
After childbirth	309	98.4
**Was your companion allowed to stay in labour room with you?**		
Yes, allowed full time	261	83.1
Yes, when needed	8	2.6
No, not allowed any time	45	14.3
**Companion with pregnant women in the labour room**		
No companion	45	14.3
In-laws (mother-in-law or sister-in-law)	140	44.6
Family (mother or sister)	82	26.1
Friends	47	15.0
**Companion during hospital stay**		
No companion	4	1.3
In-laws (mother-in-law or sister-in-law)	76	24.2
Family (mother or sister)	103	32.8
Mix (mother’s family and in-laws)	74	23.6
Misc. (friends with or without relatives)	57	18.2

**Fig 1:**
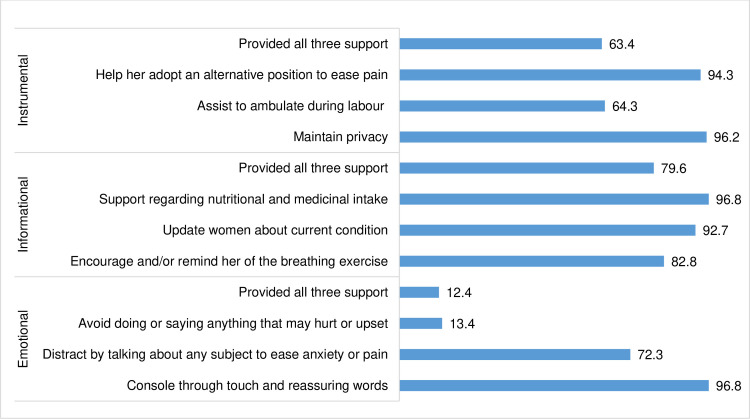
Characteristics of birth companionship.

Fig 1 illustrates that the most common types of support provided by birth companions were: informational (79.6%), followed by instrumental (63.4%), and emotional (12.4%). Furthermore, the most commonly provided supports were consoling touch and reassuring words (96.8%), supporting nutritional and medicinal needs (96.8%), and maintained privacy (96.2%). On the contrary, avoid doing or saying anything to the women that may hurt was the lowest with 13.4%. The majority of women received multiple forms of support, with a substantial proportion receiving eight out of nine possible types of support assessed (results not shown).

Table 4 presents the relationship between the level of companion support and women’s experiences of mistreatment during childbirth, as well as symptoms of postpartum anxiety and depression. Lack of companion support led to a significant increase in women’s experiences of overall mistreatment, lack of professional standards, ineffective communication, non-confidential care, non-inclusive care, and lack of supportive care. Compared with women who received high companion support, the cumulative level of mistreatment was significantly higher among women who received moderate (adjusted β = 3.68, 95% CI [1.25, 6.11]; p < 0.05) and high low (adjusted β = 8.19, 95% CI [2.53, 13.85]; p < 0.05) support. Similarly, the level of non-confidential care was significantly higher among women who received moderate companion support (adjusted β = 7.86, 95% CI [3.97, 11.75]; p < 0.01) and even more so among those who received low support (adjusted β = 19.81, 95% CI [11.53, 28.08]; p < 0.01), compared to women with high support. Furthermore, the mean score of ineffective communication was 15.49 units higher (95% CI -0.02, 30.99; p = 0.05) among women received low companion support as compared with women receiving high support. The mean score for lack of supportive care substantially increased among women who received moderate companion support (adjusted β = 16.61, 95% CI [11.93, 21.29]; p < 0.01), and this increase was even greater among those with low companion support (adjusted β = 27.32, 95% CI [14.16, 40.49]; p < 0.001), in comparison to women who received high companion support. Women who received low support from their birth companions were nearly three times (adjusted OR=2.86, 95% CI 1.52 – 5.39) more likely to experience postpartum anxiety and depression symptoms compared to those who received high support. The experiences of lack of professional standards was high among women who received low support from their companion as compared with their counterparts. Counterintuitively, we found that a lack of companion support was associated with fewer experiences of non-inclusive care. Women who received low companion support reported lower scores (adjusted β = -10.77, 95% CI [-13.05, -8.49]; p < 0.001) for non-inclusive care compared to those who received high companion support.

**Table 4:** Relationship between companion support and women’s experiences of mistreatment during childbirth and symptoms of postpartum anxiety and depression.

Characteristics	Crude	Adjusted^1^
	β (95% CI)	β (95% CI)
**SUPPORTIVE AND RESPECTFUL MATERNITY CARE (score range: 0–100)**		
	**Overall mistreatment**	
Companion support [Ref: High (Score:8–9)]	1	1
Moderate (Score: 6–7)	3.57 (0.95 - 6.19)*	3.68 (1.25 - 6.11)*
Low (Score: 0–5)	8.66 (3.71 - 13.61)**	8.19 (2.53 - 13.85)*
**RESPECTFUL MATERNITY CARE (score range: 0–100)**		
	**Physical abuse**	
Companion support [Ref: High (Score:8–9)]	1	1
Moderate (Score: 6–7)	1.45 (0.18 - 2.71)*	1.4 (-0.26 - 3.06)
Low (Score: 0–5)	0.89 (-3.1 - 4.87)	0.85 (-2.54 - 4.24)
	**Verbal abuse**	
Companion support [Ref: High (Score:8–9)]	1	1
Moderate (Score: 6–7)	1.35 (-0.71 - 3.41)	1.42 (-0.36 - 3.2)
Low (Score: 0–5)	-0.09 (-3.16 - 2.99)	-0.64 (-6.12 - 4.85)
	**Lack of professional standards**	
Companion support [Ref: High (Score:8–9)]	1	1
Moderate (Score: 6–7)	0.77 (-3.12 - 4.65)	1.66 (-1.42 - 4.74)
Low (Score: 0–5)	5.28 (0.03 - 10.54)*	5.2 (0.47 - 9.93)*
	**Ineffective communication**	
Companion support [Ref: High (Score:8–9)]	1	1
Moderate (Score: 6–7)	4.19 (-6.56 - 14.94)	3.93 (-7.05 - 14.9)
Low (Score: 0–5)	16.49 (2.46 - 30.52)*	15.49 (-0.02 - 30.99)*
	**Non-confidential care**	
Companion support [Ref: High (Score:8–9)]	1	1
Moderate (Score: 6–7)	8.04 (4.77 - 11.31)**	7.86 (3.97 - 11.75)**
Low (Score: 0–5)	20.74 (13.62 - 27.85)**	19.81 (11.53 - 28.08)**
	**Health system culture & constraints**	
Companion support [Ref: High (Score:8–9)]	1	1
Moderate (Score: 6–7)	-1.14 (-11.31 - 9.03)	-0.29 (-6.4 - 5.83)
Low (Score: 0–5)	4.63 (-8.86 - 18.12)	6.06 (-3.72 - 15.84)
**INCLUSIVE CARE (score range: 0–100)**		
	**Non-inclusive care**	
Companion support [Ref: High (Score:8–9)]	1	1
Moderate (Score: 6–7)	-8.37 (-14.64 – [-2.09])*	-8.69 (-14.97 – [-2.41])*
Low (Score: 0–5)	-10.01 (-11.95 [-8.07])***	-10.77 (-13.05 – [-8.49])***
	**Stigma and discrimination**	
Companion support [Ref: High (Score:8–9)]	1	1
Moderate (Score: 6–7)	0.58 (-0.45 - 1.61)	0.52 (-0.47 - 1.52)
Low (Score: 0–5)	-0.17 (-0.45 - 0.1)	-0.41 (-0.83 - 0.01)
**SUPPORTIVE CARE (score range: 0–100)**		
	**Lack of supportive care**	
Companion support [Ref: High (Score:8–9)]	1	1
Moderate (Score: 6–7)	16.94 (12.02 - 21.86)**	16.61 (11.93 - 21.29)**
Low (Score: 0–5)	30.17 (18.29 - 42.06)***	27.32 (14.16 - 40.49)***
**MATERNAL MENTAL HEALTH**		
	**Postpartum anxiety and depression symptoms**	
	**OR (95% CI)**	**aOR (95% CI)**
Companion support [Ref: High (Score:8–9)]	1	1
Moderate (Score: 6–7)	0.94 (0.55 - 1.62)	0.98 (0.6 - 1.6)
Low (Score: 0–5)	2.81 (1.42 - 5.57)**	2.86 (1.52 - 5.39)**

^1^Adjusted for women’s age, primigravida, mother tongue, education, household poverty, involvement in household decision making, mode of birth, antenatal care for index birth and sex of index baby

*p < 0.05, **p < 0.01, ***p < 0.001

The relationship between the three types of companion support and mistreatment, as well as symptoms of postpartum anxiety and depression, is detailed in Table 5. Women who received less than three forms of instrumental support reported significantly higher experiences of lack of supportive care (adjusted β = 11.61, 95% CI [3.74, 19.49]; p < 0.05). Similarly, lack of emotional support increased the experiences of lack of supportive care (adjusted β = 11.61, 95% CI [3.74, 19.49]; p < 0.05) and ineffective communication (adjusted β = 8.46, 95% CI [4.81, 12.12]; p < 0.01).

**Table 5:** Relationship between type of companion support and women’s experiences of mistreatment during childbirth and symptoms of postpartum anxiety and depression.

Characteristics	Instrumental support		Emotional support		Informational support	
	Crude	Adjusted^1^	Crude	Adjusted^1^	Crude	Adjusted^1^
	β (95% CI)	β (95% CI)	β (95% CI)	β (95% CI)	β (95% CI)	β (95% CI)
**SUPPORTIVE AND RESPECTFUL MATERNITY CARE (score range: 0–100)**						
	**Overall mistreatment**					
All three support	1	1	1	1	1	1
Less than three support	2.13 (-0.06 - 4.32)	1.88 (-0.2 - 3.96)	3.2 (0.31 - 6.09)*	2.8 (-0.65 - 6.25)	8.43 (4.55 - 12.31)**	8.12 (3.74 - 12.5)**
**RESPECTFUL MATERNITY CARE (score range: 0–100)**						
	**Physical abuse**					
All three support	1	1	1	1	1	1
Less than three support	1 (-0.49 - 2.5)	0.95 (-0.44 - 2.34)	0.78 (-1 - 2.56)	0.96 (-1.05 - 2.96)	1.34 (-0.46 - 3.14)	1.27 (-0.28 - 2.81)
	**Verbal abuse**					
All three support	1	1	1	1	1	1
Less than three support	0.13 (-1.98 - 2.25)	-0.12 (-1.49 - 1.24)	0.77 (-4.09 - 5.64)	0.87 (-5.67 - 7.42)	2.68 (0.14 - 5.22)*	2.08 (-1.78 - 5.94)
	**Lack of professional standards**					
All three support	1	1	1	1	1	1
Less than three support	1.83 (-1.26 - 4.92)	1.63 (-1.31 - 4.56)	-2.26 (-10.29 - 5.76)	-1.8 (-8.93 - 5.33)	5.87 (-0.82 - 12.55)	6.11 (0.02 - 12.19)*
	**Ineffective communication**					
All three support	1	1	1	1	1	1
Less than three support	0.95 (-6.71 - 8.61)	0.2 (-6.56 - 6.95)	10.2 (5.22 - 15.17)**	8.46 (4.81 - 12.12)**	16.06 (0.69 - 31.43)*	15.77 (0.06 - 31.48)*
	**Non-confidential care**					
All three support	1	1	1	1	1	1
Less than three support	2.08 (-0.99 - 5.16)	1.44 (-1.82 - 4.69)	6.85 (0.94 - 12.76)*	5.43 (-2.58 - 13.45)	16.83 (12.05 - 21.62)***	15.9 (10.02 - 21.78)**
	**Health system culture & constraints**					
All three support	1	1	1	1	1	1
Less than three support	-0.38 (-8.62 - 7.86)	-0.24 (-7.24 - 6.76)	-2.43 (-12.91 - 8.05)	-0.1 (-3.83 - 3.63)	3.73 (-5.02 - 12.47)	6.04 (-2.42 - 14.51)
**INCLUSIVE CARE (score range: 0–100)**						
	**Non-inclusive care**					
All three support	1	1	1	1	1	1
Less than three support	-4.17 (-9.24 - 0.9)	-4.08 (-8.81 - 0.64)	-4.55 (-12.48 - 3.39)	-4.86 (-13.95 - 4.24)	-7.29 (-10.64 - -3.94)**	-7.77 (-11.16 - -4.37)**
	**Stigma and discrimination**					
All three support	1	1	1	1	1	1
Less than three support	0.41 (-0.85 - 1.66)	0.37 (-0.78 - 1.52)	0 (-0.55 - 0.55)	-0.1 (-0.91 - 0.72)	0.66 (-1.34 - 2.67)	0.38 (-1.16 - 1.92)
**SUPPORTIVE CARE (score range: 0–100)**						
	**Lack of supportive care**					
All three support	1	1	1	1	1	1
Less than three support	12.91 (4.67 - 21.14)*	11.61 (3.74 - 19.49)*	14.91 (9.77 - 20.05)**	11.46 (3.49 - 19.42)*	28.69 (21.37 - 36)***	25.49 (18 - 32.97)***
**MATERNAL MENTAL HEALTH**						
	**Postpartum anxiety and depression symptoms**					
All three support	1	1	1	1	1	1
Less than three support	1.55 (0.89 - 2.71)	1.64 (0.97 - 2.8)	0.76 (0.35 - 1.65)	0.74 (0.32 - 1.67)	2.19 (1.19 - 4.04)*	2.12 (1.03 - 4.35)*

^1^Adjusted for women’s age, primigravida, mother tongue, education, household poverty, involvement in household decision making, mode of birth, antenatal care for index birth and sex of index baby

*p < 0.05, **p < 0.01, ***p < 0.001

Lack of informational support was associated with overall mistreatment (adjusted β = 8.12, 95% CI [3.74, 12.5]; p < 0.01), including specific types of mistreatment: lack of professional standards (adjusted β = 6.11, 95% CI [0.02, 12.19]; p < 0.05), ineffective communication (adjusted β = 15.77, 95% CI [0.06, 31.48]; p < 0.05), non-confidential care (adjusted β = 15.9, 95% CI [10.02, 21.78]; p < 0.01), and lack of supportive care (adjusted β = 25.49, 95% CI [18.0, 32.97]; p < 0.001). Notably, the odds of postpartum anxiety and depressive symptoms were 2.21 times higher (95% CI [1.03, 4.35]) among women who did not receive all three forms of informational support from their companion compared to those who did.

**Fig 2:**
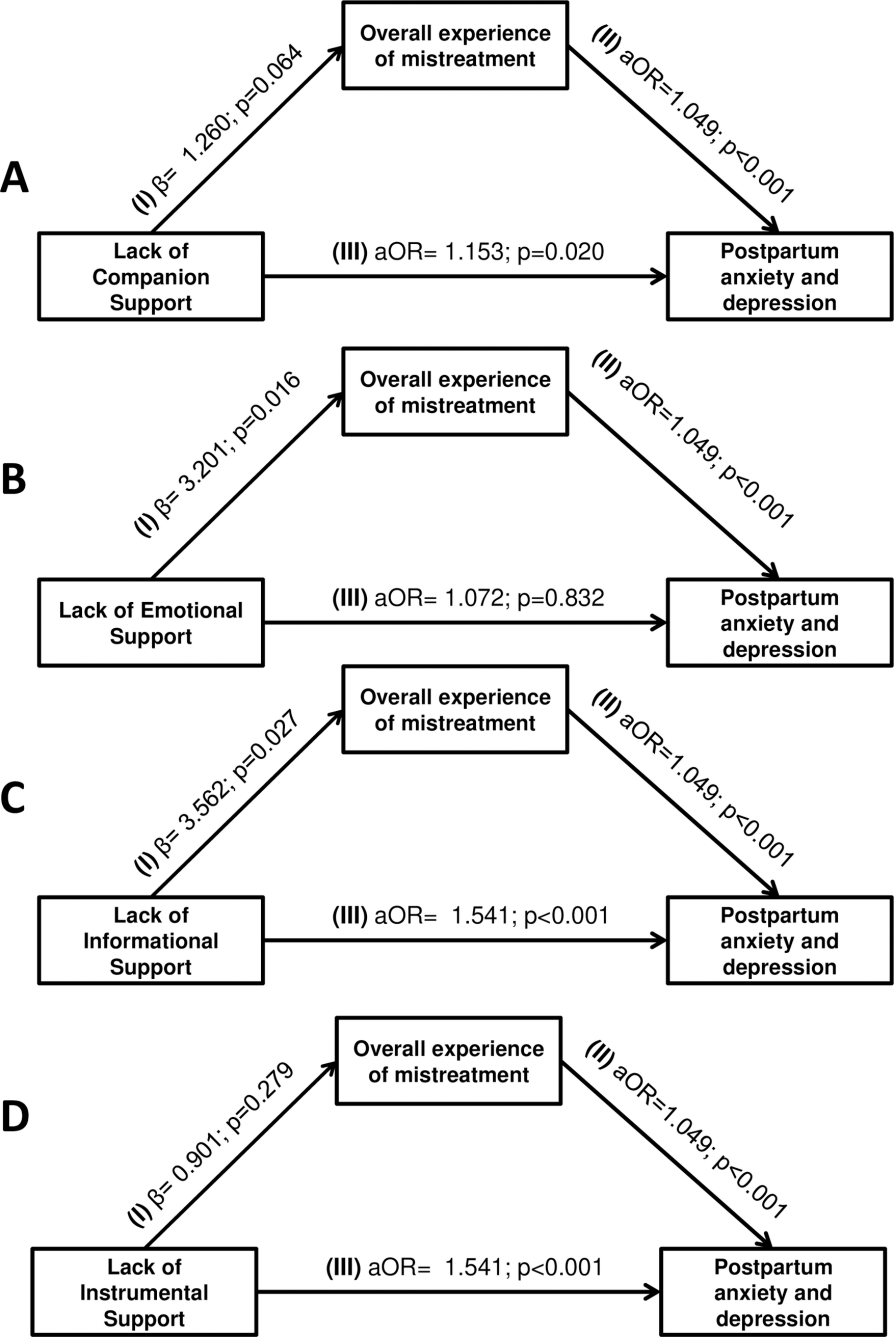
A-D: Links between companionship, mistreatment, and postpartum anxiety and depression.

Results from the mediation analysis revealed that companion support has both direct and indirect effects on symptoms of postpartum anxiety and depression, which are mediated by experiences of mistreatment during childbirth. For every one-unit decrease in companion support, the likelihood of postpartum anxiety and depression symptoms increases by 15.3% (aOR = 1.15, 95% CI: 1.02–1.30; p = 0.020). The indirect path revealed that a lack of companion support increases (marginally significant) the likelihood of experiencing mistreatment during childbirth (β = 1.26, 95% CI -0.10 – 2.62; p = 0.064), which in turn raises the odds of postpartum anxiety and depression symptoms by 1.05 times (95% CI 1.02 - 1.08; p < 0.001) for each unit increase in mistreatment (Fig 2A).

Similarly, lack of informational support shows both direct and indirect effects. A one-unit decrease in informational support increases the likelihood of anxiety and depression symptoms by 54.1% (aOR = 1.54, 95% CI 1.32 - 1.80; p < 0.001) and is linked to a 3.53-unit increase in mistreatment (β = 3.56, 95% CI 0.61 – 6.51; p = 0.027). The increased level of mistreatment was associated with higher odds of postpartum anxiety and depression symptoms. Emotional support exhibited only an indirect effect where reduced emotional support is associated with increased mistreatment (β = 3.20, 95% CI 0.91 - 5.49, p = 0.016), which in turn raises the likelihood of anxiety and depression symptoms 4.9% per unit increase in mistreatment (aOR = 1.049, 95% CI 1.02 - 1.08; p < 0.001). In contrast, instrumental support has a direct effect, with a one-unit decrease increasing the likelihood of anxiety and depression symptoms by 42.0% (aOR = 1.42, 95% CI 1.15 - 1.75; p = 0.001).

## Discussion

Despite the recommendation from the WHO of allowing women to choose a personal birth companion, the policy is not widely implemented across many LMICs [8]. While research shows that having a personal birth companion can reduce mistreatment during childbirth, there is limited evidence on how the characteristics of the companionship affect mistreatment and maternal mental health. We examined the characteristics of support provided by birth companions and its association with women’s experiences of mistreatment and symptoms of postpartum anxiety and depression.

Our study found that the vast majority of women were accompanied by a personal companion during labour, childbirth, and post-delivery, typically in-laws and family members who provided substantial informational, emotional, and practical support. Higher levels of companion support were significantly associated with reduced mistreatment, particularly in communication, respect for confidentiality, and supportive care. Women who received moderate to high levels of support experienced less mistreatment and overall better care compared to those who received low support. In addition, greater companion support was associated with reduced postpartum anxiety and depression symptoms, both directly and indirectly, by decreasing experiences of mistreatment during childbirth..

The presence of birth companions in our study is higher compared to other South Asian countries [46], including Bangladesh (55.5%) [47] and India (73%) [48]. However, it aligns with findings from a previous study conducted in Pakistan [49]. A likely reason for this higher presence is that our analysis is based on a post-intervention survey where birth companionship was a core component of the intervention [31,33,50]. Moreover, companion engagement was perceived as an effective task-sharing strategy that allowed maternity service providers to offer continuous support, thereby reducing their workload [50–52]. In our study, the most common companions were in-laws and other family members, excluding husbands. Although husbands were present at the hospital, they were not permitted in maternity wards, including labour rooms, due to restrictive policies [28], socio-cultural norms [29,30], and privacy concerns for other birthing women, as public hospitals often manage multiple women in large labour rooms [29].

Consistent with other studies [12,14,15,53], we observed a strong relationship between birth companionship and reduced mistreatment. Specifically, experiences of non-confidential care, ineffective communication, and lack of supportive care were significantly reduced. Of the three different types of companion support, informational support emerged as a stronger determinant of the experiences of mistreatment and symptoms of anxiety and depression during postpartum. Qualitative studies also reveal that companions play a crucial role in providing physical, informational, and emotional support to birthing women [10]. For instance, companions help bridge communication gaps by explaining technical jargon used by healthcare providers and providing timely information about labour progress. They also assist in maintaining privacy during physical examinations [14,30] and are well-positioned to offer psychosocial support, understanding the emotional needs of birthing women [50,54]. In terms of informational support, two psychological factors—women’s internal locus of control and increased trust in their companion—may explain the link between informational support, mistreatment, and postpartum depression. Labour and childbirth can be distressing and create uncertainty. Informational support, which provides facts, knowledge, or advice, can alleviate this [55]. When companions offer updates on the mother’s health, it may boost her confidence and internal locus of control [56,57], fostering a sense of control and encouraging active participation in the birthing process. Furthermore, informational support may strengthen women’s trust in their companions, reassuring them that they have someone connected with the healthcare staff [30]. This support could help prevent the risk of mistreatment and hence postnatal anxiety and depression.

Our study provides evidence that overall support from birth companions, particularly informational support, has a direct effect on reducing anxiety and depression symptoms and an indirect effect through the reduction of mistreatment during childbirth. These findings emphasise the critical role of birth companions not only in addressing mistreatment but also in enhancing maternal mental health. The association between birth companionship and mistreatment aligns with findings from other studies [20,21,58]. While limited evidence exists on the exact pathways through which companionship improves mental health, a plausible explanation is that support during facility-based childbirth acts as a buffer against psychological stressors [59], reducing stress hormones and improving labour physiology and emotional well-being [60–62]. This support positively influences the fetopelvic relationship, facilitating the birthing process [63], decreasing mistreatment, reducing medical interventions, and lowering the risk of complications. Consequently, companion support leads to better birthing outcomes, greater maternal satisfaction, and improved postpartum mental health. Findings from our path analysis further substantiate the proposed mechanism linking companionship with maternal mental health. Further research with larger sample sizes is needed to confirm this mediation pathway.

Our study has several strengths and limitations. Among the strengths, we adapted validated tools to measure mistreatment and used a locally validated tool to screen symptoms of postpartum anxiety and depression. In addition, the inclusion of path analysis adds unique value by explaining the mechanisms linking companionship with maternal mental health. Items for companion support were developed based on WHO recommendations, cultural practices, and input from maternity staff in labour room settings. These indicators were identified through a systematic, iterative process to ensure content and cultural relevance. The study’s limitations include its cross-sectional design, which restricts us from drawing causal inferences about the relationship between birth companionship and maternal mental health. Furthermore, while the term ‘effect’ is used in the context of path analysis to describe associations, the cross-sectional nature of the dataset and the lack of an established theoretical framework for these relationships preclude any causal inferences. The study was conducted in secondary-level hospitals that offer basic emergency obstetric and newborn care because this is the first level of the local health system where formal labour room and ward facilities are offered; therefore, the findings may have limited generalisability to other settings such as primary or tertiary care level. Although we developed a comprehensive measure of birth companionship, it has not been validated. The analysis is based on data from the endline survey of a larger experimental study in which birth companionship was a key component of the intervention. Therefore, estimates of the increased presence and support of companions should be interpreted cautiously when compared to observational studies.

## Conclusion

Our study findings indicate a strong positive relationship between birth companionship and women’s experiences of mistreatment, particularly regarding non-confidential care, lack of supportive care, and ineffective communication. We also found that support from birth companions, particularly informational support, has a direct effect on reducing anxiety and depression symptoms and an indirect effect through the reduction of mistreatment during childbirth. Of the three types of companion support, informational support emerged as a stronger determinant that has a protective effect on both experiences of mistreatment and symptoms of postpartum anxiety and depression. These findings highlight the critical role of personal companions during facility-based childbirth in reducing mistreatment and improving maternal mental health. There is a need for supportive policies and health system interventions that actively encourage the engagement of companions from a woman’s personal network during labour and childbirth.

## Supporting information

S1 Checklist Inclusivity in global research. (DOCX)
